# Supplementary material for: Joint Effect of Multiple Metals on Hyperuricemia and Their Interaction with Obesity: A Community-Based Cross-Sectional Study in China
Source: Nutrients. 2023 Jan 20;15(3):552. doi: 10.3390/nu15030552 (PMC9921062; doi:10.3390/nu15030552)
Supplement: Supplementary file 1 [file nutrients-15-00552-s001.zip › Supplementary figure.pdf]

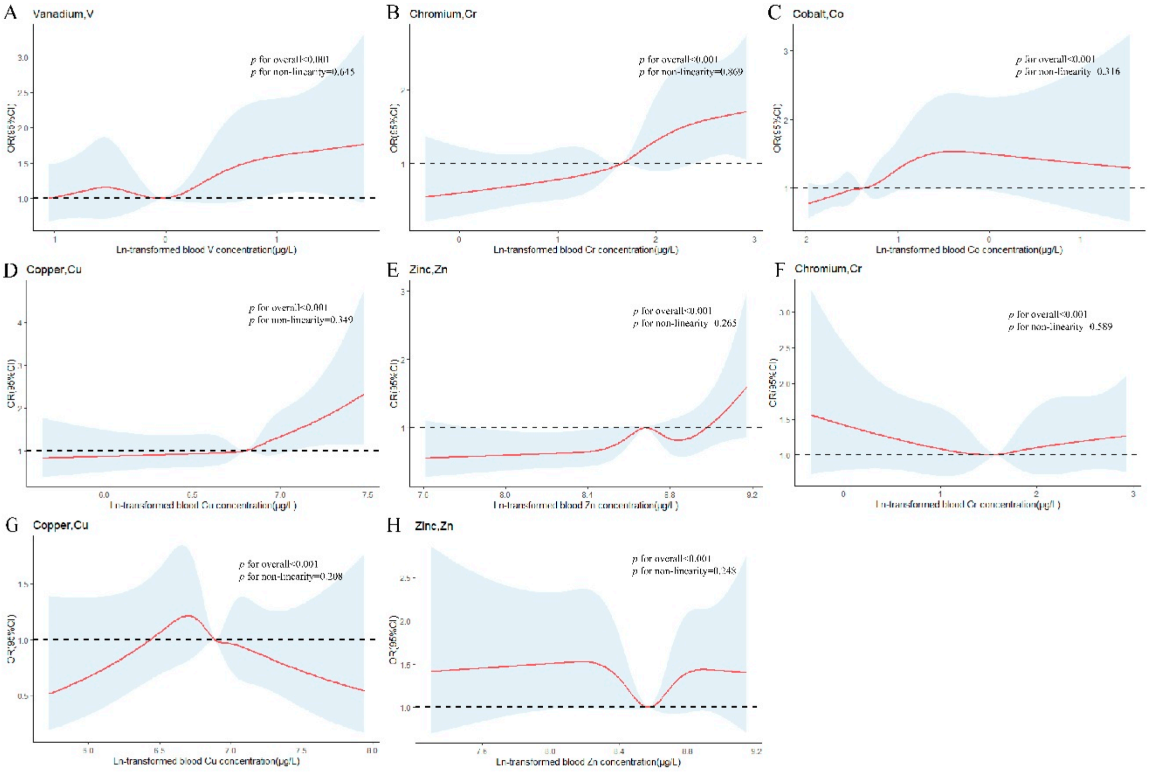


**Figure S1.** The RCS with five knots for the association between blood metal levels and incident hyperuricemia. The red lines indicate adjusted odds ratios, and blue areas represent 95% confidence intervals based on the restricted cubic spline models for the natural log-transformed concentrations of whole blood V, Cr, Co, Cu, Zn and As in both gender(A-E for male and F-H for female). The reference values were set at the 10th percentiles. Adjusted variables were the same as listed for single-metal model.
