# Supplementary material for: Joint Effect of Multiple Metals on Hyperuricemia and Their Interaction with Obesity: A Community-Based Cross-Sectional Study in China
Source: Nutrients. 2023 Jan 20;15(3):552. doi: 10.3390/nu15030552 (PMC9921062; doi:10.3390/nu15030552)
Supplement: Supplementary file 1 [file nutrients-15-00552-s001.zip › Supplementary table.pdf]

Table S1. Analysis results of Seronorm Trace Elements Whole blood L-1and L-2 (μg/L).

Whole blood metals (μg/L)	L-1			L-2		
	Measured value mean	Median (Reference range)	RSDs [a] /%	Measured value mean	Median (Reference range)	RSDs/%
Vanadium (V)	1.10	0.97 (0.58, 1.35)	4.21	3.35	3.10 (2.40, 3.70)	3.13
Chrome (Cr)	0.57	0.45 (0.27, 0.63)	5.67	10.80	10.00 (8.00, 12.00)	5.42
Manganese (Mn)	19.80	18.40 (14.70, 22.10)	4.47	25.40	24.20 (22.20, 26.10)	3.31
Cobalt (Co)	0.17	0.20 (0.12, 0.28)	3.90	5.16	5.00 (4.00, 6.00)	3.56
Nickel (Ni)	1.35	1.38 (1.10,1.66)	4.51	9.50	9.20 (7.30, 11.00)	3.37
Copper (Cu)	0.66	0.64 (0.51, 0.76)	4.84	0.97	0.98 (0.89, 1.06)	4.65
Zinc (Zn)	4.82	4.30 (3.40, 5.20)	2.50	5.96	5.80 (4.80, 6.80)	1.57
Arsenic (As)	5.14	4.60 (3.70, 5.50)	5.37	13.60	12.20 (9.80, 14.70)	4.92
Selenium (Se)	68.90	60.00 (48.00, 72.00)	6.98	161.00	144.00 (113.00, 175.00)	5.85
Molybdenum (Mo)	0.53	0.51 (0.41, 0.61)	4.80	5.23	4.50 (3.60, 5.40)	4.38
Cadmium (Cd)	0.31	0.28 (0.17, 0.40)	5.23	5.70	5.10 (4.10, 6.10)	5.46
Thallium (Tl)	10.20	7.00 (3.00, 11.00)	8.98	11.40	10.10 (8.10, 12.10)	4.45
Lead (Pb)	9.60	9.90 (7.90, 11.90)	2.93	310.00	303.00 (272.00, 334.00)	2.77

[a] RSD, relative standard deviation.

Table S2. Limits of detection, percentages of samples below detection limits (n=3029).

Whole blood metals (μg/L)	LOD	Total No. (%) <LOD [a]
V	0.5	710 (23.44)
Cr	1	49 (1.62)
Mn	0.5	10 (0.33)
Co	0.2	881 (29.09)
Ni	0.8	506 (16.71)
Cu	10	0 (0.00)
Zn	20	0 (0.00)
As	1	183 (6.04)
Se	2	63 (2.08)
Mo	1	1455 (48.04)
Cd	0.2	44 (1.45)
Tl	0.2	2692 (88.87)
Pb	20	427 (14.10)

[a] LOD, limit of detection.

Table S3. Baselines characteristic of different gender participants in study.

Variables [a]	Male		*p* - value	Female		*p*- value
	No hyperuricemia (n=939)	Hyperuricemia (n=433)		No hyperuricemia (n=1336)	Hyperuricemia (n=321)	
Age (years)	53.70±0.50	53.56±0.73	0.870	50.76±0.40	56.28±0.78	<0.001*
Residence area (%)			0.673			0.891
Coastal	509 (54.2)	240 (55.4)		731 (54.7)	177 (55.1)	
Inland	430 (45.8)	193 (44.6)		605 (45.3)	144 (44.9)	
Education level (%)			0.031*			0.370
Below high school	669 (71.2)	278 (64.2)		1025 (76.7)	258 (80.4)	
High school	183 (19.5)	107 (24.7)		180 (13.5)	37 (11.5)	
Over high school	87 (9.3)	48 (11.1)		131 (9.8)	26 (8.1)	
Alcohol use (%)			0.032*			0.146
Drinker	473 (50.4)	245 (56.6)		326 (24.4)	66 (20.6)	
Non-drinker	466 (49.6)	188 (43.4)		1010 (75.6)	255 (79.4)	
Smoking status (%)			0.013*			0.309
Current smokers	529 (56.3)	225 (52.0)		33 (2.5)	7 (2.1)	
Former smokers	130 (13.8)	46 (10.6)		12 (0.9)	6 (1.9)	
Nonsmokers	280 (29.8)	162 (37.4)		1291 (96.6)	308 (96.0)	
Sitting time (%)			0.507			0.752
< 4 h/day	353 (37.6)	151 (34.9)		572 (42.8)	133 (41.4)	
4 to <6 h/day	264 (28.1)	123 (28.4)		379 (28.4)	89 (27.7)	
6 to <8 h/day	147 (15.7)	81 (18.7)		177 (13.2)	41 (12.8)	
≥8 h/day	175 (18.6)	78 (18.0)		208 (15.6)	58 (18.1)	
Physical activity (%)			0.035*			0.894
Yes	179 (19.1)	104 (24)		200 (15.0)	49 (15.3)	
No	760 (80.9)	329 (76.0)		1136 (85.0)	272 (84.7)	

WHt R	0.49 (0.45, 0.53)	0.51 (0.47, 0.56)	<0.001*	0.51 (0.46, 0.54)	0.54 (0.50, 0.58)	<0.001*
BMI (kg/m^2)	22.71 (20.57, 24.98)	24.13 (21.77, 26.42)	<0.001*	22.58 (20.67, 24.86)	24.40 (22.53, 26.96)	<0.001*
UA (μmol/L)	341.80 (302.90, 379.10)	476.00 (443.90, 518.70)	<0.001*	275.10 (237.50, 311.80)	405.00 (381.90, 441.30)	<0.001*
Log_2UA	8.38±0.01	8.93±0.01	<0.001*	8.07±0.01	8.71±0.01	<0.001*
TC (mmol/L)	4.94 (4.29, 5.54)	5.17 (4.51, 5.93)	<0.001*	5.02 (4.38, 5.67)	5.32 (4.62, 5.99)	<0.001*
TG (mmol/L)	1.04 (0.72, 1.52)	1.51 (1.05, 2.17)	<0.001*	0.92 (0.67, 1.38)	1.31 (0.89, 2.02)	<0.001*
Diabetes mellitus (%)			0.935			<0.001*
Yes	79 (8.4)	37 (8.5)		97 (7.3)	46 (14.3)	
No	860 (91.6)	396 (91.5)		1239 (92.7)	275 (85.7)	
Hypertension (%)			<0.001*			<0.001*
Yes	321 (34.2)	203 (46.9)		409 (30.6)	157 (48.9)	
No	618 (65.8)	230 (53.1)		927 (69.4)	164 (51.1)	
Central obesity (%)			<0.001*			<0.001*
Yes	337 (35.9)	212 (49.0)		497 (37.2)	199 (62.0)	
No	602 (64.1)	221 (51.0)		839 (62.8)	122 (38.0)	
Blood metals (μg/L)						
V	0.91 (0.35, 1.44)	1.06 (0.53, 1.74)	0.003*	0.97 (0.53, 1.54)	0.95 (0.35, 1.5)	0.174
Cr	4.97 (3.74, 6.52)	5.42 (4.18, 8.24)	<0.001*	4.80 (3.70, 6.03)	4.93 (3.55, 6.32)	0.634
Mn	13.10 (10.00, 16.64)	14.01 (10.81, 17.55)	0.003*	14.75 (11.43, 18.74)	14.24 (10.75, 18.66)	0.493
Co	0.24 (0.14, 0.34)	0.26 (0.14, 0.36)	0.038*	0.32 (0.21, 0.45)	0.30 (0.21, 0.38)	0.023*
Ni	2.16 (1.19, 3.95)	2.32 (1.16, 3.94)	0.915	2.29 (1.35, 4.11)	2.06 (1.27, 3.88)	0.306
Cu	891.83 (807.64, 997.45)	918.81 (823.25, 1019.56)	0.028*	981.39 (879.45, 1102.37)	986.14 (888.15, 1091.75)	0.679
Zn	5832.53 (5069.59, 6721.29)	5984.06 (5272.15, 6914.89)	0.007*	5282.29 (4536.17, 6142.88)	5343.35 (4531.62, 6325.64)	0.393
As	4.45 (2.74, 7.13)	5.10 (2.74, 7.91)	0.019*	4.18 (2.46, 6.61)	4.47 (2.87, 7.33)	0.040*
Se	166.04 (138.61, 194.85)	170.28 (138.97, 204.31)	0.164	163.74 (136.89, 192.60)	166.18 (143.46, 197.30)	0.017*
Mo	1.01 (0.71, 2.05)	1.04 (0.71, 2.20)	0.564	1.06 (0.71, 2.05)	1.15 (0.71, 2.36)	0.130

Cd	3.12 (1.62, 5.82)	2.93 (1.29, 5.79)	0.132	1.91 (1.19, 3.30)	2.07 (1.23, 3.13)	0.805
Tl	0.14 (0.14, 0.14)	0.14 (0.14, 0.14)	0.208	0.14 (0.14, 0.14)	0.14 (0.14, 0.14)	0.822
Pb	47.46 (32.35, 67.95)	46.76 (32.34, 70.16)	0.812	33.13 (22.56, 50.07)	35.93 (25.83, 52.69)	0.006*

BMI, body mass index; TC, total cholesterol; TG, triglycerides; UA, uric acid; WHt R, waist height ratio.

[a] Values are presented as n(%), mean ± SD or median (IQR)

*$p < 0.05$

Table S4. The correlations between whole blood metals among the study population.

	V	Cr	Mn	Co	Ni	Cu	Zn	As	Se	Mo	Cd	Tl	Pb
Male													
V	1	0.539*	0.085*	0.033	0.081*	0.116*	-0.028	0.175*	-0.252*	0.090*	-0.129*	0.256*	-0.101*
Cr		1	0.233*	0.081*	0.083*	0.132*	0.143*	0.161*	-0.058*	0.108*	-0.076*	0.116*	-0.071*
Mn			1	0.264*	0.134*	0.178*	0.220*	0.164*	0.138*	0.042	0.032	-0.023	0.084*
Co				1	0.157*	0.059*	-0.018	0.128*	0.016	0.107*	0.001	0.028	-0.012
Ni					1	0.141*	0.168*	0.031	-0.010	0.139*	-0.005	0.053*	0.027
Cu						1	0.338*	0.017	0.122*	0.103*	0.043	0.009	0.042
Zn							1	0.085*	0.089*	0.064*	0.141*	0.074*	0.073*
As								1	-0.398*	0.225*	0.040	0.527*	-0.070*
Se									1	-0.177*	-0.032	-0.904*	0.337*
Mo										1	0.066*	0.225*	-0.062*
Cd											1	0.089*	0.354*
Tl												1	-0.279*
Pb													1
Female													
V	1	0.525*	0.075*	0.045	0.107*	0.161*	-0.033	0.161*	-0.191*	0.073*	-0.164*	0.222*	-0.121*
Cr		1	0.207*	0.098*	0.070*	0.113*	0.141*	0.126*	-0.023	0.046	-0.114*	0.113*	-0.054*
Mn			1	0.327*	0.132*	0.232*	0.250*	0.158*	0.185*	-0.013	0.133*	-0.008	0.100*

Co	1	0.135*	0.065*	-0.035*	0.133*	0.086*	0.079*	0.092*	0.000	-0.036
Ni		1	0.187*	0.155*	0.070*	0.007	0.183*	0.074*	0.065*	0.088*
Cu			1	0.316*	-0.016	0.189*	0.178*	0.011	-0.008	0.057*
Zn				1	0.068*	0.133*	0.089*	0.180*	0.077*	0.137*
As					1	-0.314*	0.065*	0.064*	0.456*	-0.018
Se						1	-0.004	-0.038	-0.838*	0.214*
Mo							1	0.087*	0.058*	0.107*
Cd								1	0.146*	0.379*
Tl									1	-0.141*
Pb										1

*p <0.05

Table S5. Adjusted odds ratio for incident hyperuricemia risk in subgroups stratified by smoking status.

	Q1	Q2	Q3	Q4	*p*- trend [a]
**Male**					
Cr (μg/L)	≤3.84	3.85-5.11	5.12-6.84	≥6.85	
Hyperuricemia/Total	92/338	93/344	110/343	138/341	
Current smokers	Ref	0.98 (0.57, 1.67)	1.30 (0.77, 2.18)	1.72 (1.05, 2.80)*	0.013*
Former smokers	Ref	0.67 (0.13, 3.40)	1.64 (0.39, 6.93)	2.61 (0.74, 9.21)	0.052
Nonsmokers	Ref	0.87 (0.44, 1.70)	0.75 (0.37, 1.49)	1.75 (0.89, 3.42)	0.119
Co (μg/L)	≤0.14	0.15-0.25	0.26-0.34	≥0.35	
Hyperuricemia/Total	140/493	72/218	102/321	119/340	
Current smokers	Ref	1.19 (0.71, 2.01)	1.13 (0.72, 1.77)	1.23 (0.78, 1.94)	0.386
Former smokers	Ref	0.28 (0.05, 1.77)	1.89 (0.55, 6.45)	3.05 (1.01, 9.25)*	0.017*
Nonsmokers	Ref	2.18 (1.11, 4.27)*	1.70 (0.90, 3.21)	2.59 (1.42, 4.74)*	0.004*
Cu (μg/L)	≤811.13	811.14-898.60	898.61-1005.97	≥1005.98	
Hyperuricemia/Total	99/343	99/343	113/343	122/343	
Current smokers	Ref	0.88 (0.54, 1.46)	0.88 (0.53, 1.46)	0.99 (0.60, 1.65)	0.974
Former smokers	Ref	0.17 (0.04, 0.84)*	0.93 (0.26, 3.33)	1.67 (0.40, 6.97)	0.161
Nonsmokers	Ref	0.93 (0.49, 1.74)	1.78 (0.95, 3.34)	2.02 (1.05, 3.88)*	0.011*
Zn (μg/L)	≤5141.11	5141.12-5906.57	5906.58-6781.64	≥6781.65	
Hyperuricemia/Total	91/343	113/343	103/343	126/343	
Current smokers	Ref	1.73 (1.04, 2.90)*	1.21 (0.71, 2.05)	1.66 (0.99, 2.79)	0.203
Former smokers	Ref	1.52 (0.37, 6.17)	0.84 (0.23, 3.06)	1.99 (0.53, 7.51)	0.491
Nonsmokers	Ref	1.21 (0.65, 2.24)	1.16 (0.61, 2.21)	1.11 (0.57, 2.19)	0.765
As (μg/L)	≤2.74	2.75-4.60	4.61-7.31	≥7.31	
Hyperuricemia/Total	109/344	88/343	111/342	125/343	
Current smokers	Ref	0.97 (0.59, 1.60)	1.32 (0.81, 2.14)	1.27 (0.77, 2.11)	0.223
Former smokers	Ref	0.31 (0.09, 1.06)	0.23 (0.07, 0.78)*	0.37 (0.08, 1.75)	0.066
Nonsmokers	Ref	0.56 (0.30, 1.07)	0.85 (0.44, 1.66)	1.39 (0.70, 2.77)	0.222
**Female**					
V (μg/L)	≤0.53	0.54-0.98	0.99-1.55	≥1.56	
Hyperuricemia/Total	97/408	63/407	79/408	77/407	
Current smokers	Ref	NA	NA	NA	NA
Former smokers	Ref	NA	NA	NA	NA
Nonsmokers	Ref	0.52 (0.35, 0.76)*	0.61 (0.41, 0.92)*	0.68 (0.47, 1.00)*	0.107

Model was adjusted for age, education level, residence area, alcohol use, physical activity, sitting time, BMI, diabetes mellitus, hypertension, TC, TG, metals in the multiple-metal model.

[a] *p*- trend across quartiles of metals was obtained by including the median of each quartile (natural log-transformed metals concentration) as a continuous variable in the model.

*p <0.05

Table S6. Adjusted odds ratio for incident hyperuricemia risk in subgroups stratified by alcohol use.

	Q1	Q2	Q3	Q4	*p*- trend [a]
**Male**					
Cr (μg/L)	≤3.84	3.85-5.11	5.12-6.84	≥6.85	
Hyperuricemia/Total	92/338	93/344	110/343	138/341	
Drinker	Ref	0.84 (0.49, 1.46)	0.98 (0.58, 1.65)	1.66 (1.01, 2.75)*	0.026*
Non-drinker	Ref	1.13 (0.64, 1.99)	1.24 (0.69, 2.23)	1.91 (1.11, 3.30)*	0.016*
Co (μg/L)	≤0.14	0.15-0.25	0.26-0.34	≥0.35	
Hyperuricemia/Total	140/493	72/218	102/321	119/340	
Drinker	Ref	1.25 (0.74, 2.11)	1.62 (1.02, 2.56)*	1.81 (1.14, 2.89)*	0.006*
Non-drinker	Ref	1.27 (0.72, 2.23)	1.08 (0.65, 1.81)	1.48 (0.91, 2.41)	0.173
Cu (μg/L)	≤811.13	811.14-898.60	898.61-1005.97	≥1005.98	
Hyperuricemia/Total	99/343	99/343	113/343	122/343	
Drinker	Ref	0.79 (0.49, 1.30)	1.08 (0.65, 1.79)	1.27 (0.76, 2.12)	0.196
Non-drinker	Ref	0.88 (0.50, 1.53)	1.26 (0.74, 2.14)	1.38 (0.80, 2.38)	0.124
Zn (μg/L)	≤5141.11	5141.12-5906.57	5906.58-6781.64	≥6781.65	
Hyperuricemia/Total	91/343	113/343	103/343	126/343	
Drinker	Ref	1.38 (0.83, 2.28)	1.06 (0.64, 1.76)	1.24 (0.73, 2.12)	0.719
Non-drinker	Ref	1.56 (0.92, 2.64)	1.01 (0.58, 1.78)	1.45 (0.85, 2.46)	0.375
As (μg/L)	≤2.74	2.75-4.60	4.61-7.31	≥7.31	
Hyperuricemia/Total	109/344	88/343	111/342	125/343	
Drinker	Ref	0.68 (0.41, 1.11)	0.94 (0.59, 1.51)	1.00 (0.60, 1.67)	0.820
Non-drinker	Ref	0.79 (0.46, 1.36)	0.97 (0.55, 1.71)	1.60 (0.91, 2.81)	0.086
**Female**					
V (μg/L)	≤0.53	0.54-0.98	0.99-1.55	≥1.56	
Hyperuricemia/Total	97/408	63/407	79/408	77/407	
Drinker	Ref	0.52 (0.21, 1.33)	0.54 (0.20, 1.46)	0.56 (0.22, 1.44)	0.333
Non-drinker	Ref	0.56 (0.37, 0.85)*	0.69 (0.44, 1.07)	0.70 (0.47, 1.06)	0.176

Model was adjusted for age, education level, residence area, smoking status, physical activity, sitting time, BMI, diabetes mellitus, hypertension, TC, TG, metals in the multiple-metal model.

[a] *p*- trend across quartiles of metals was obtained by including the median of each quartile (natural log-transformed metals concentration) as a continuous variable in the model.

**p* <0.05.

Table S7. Odds ratios and 95% confidence intervals for hyperuricemia according to the combined categories of whole blood metal concentrations in male.

	n (hyperuricemia / non-hyperuricemia)	OR (95%CI)	OR- int [a]	RERI [b] (95%CI)	AP [b] (95%CI)	S [b] (95%CI)
Cr-Co			1.01 (0.62, 1.65)	0.16 (-0.47, 0.78)	0.08 (-0.25, 0.41)	1.21 (0.53, 2.79)
Low Cr + Low Co	91/272	Ref				
Low Cr + High Co	93/230	1.33 (0.93, 1.92)				
High Cr + Low Co	111/212	1.41 (0.99, 2.01)				
High Cr + High Co	138/225	1.90 (1.35, 2.68)*				
Cr-Zn			1.55 (0.94, 2.55)	0.49 (0.03, 0.95)*	0.34 (0.02, 0.65)*	NA
Low Cr + Low Zn	110/289	Ref				
Low Cr + High Zn	74/213	0.83 (0.57, 1.19)				
High Cr + Low Zn	94/193	1.14 (0.80, 1.62)				
High Cr + High Zn	155/244	1.45 (1.05, 2.01)*				
Cr-As			1.20 (0.73, 1.96)	0.34 (-0.24, 0.92)	0.19 (-0.13, 0.51)	1.76 (0.49, 6.39)
Low Cr + Low As	89/262	Ref				
Low Cr + High As	95/240	1.16 (0.80, 1.67)				
High Cr + Low As	108/227	1.29 (0.91, 1.84)				
High Cr + High As	141/210	1.79 (1.24, 2.57)*				
Cr-Cu			0.96 (0.59, 1.57)	0.09 (-0.56, 0.73)	0.05 (-0.29, 0.39)	1.11 (0.5, 2.46)
Low Cr + Low Cu	90/272	Ref				
Low Cr + High Cu	94/230	1.36 (0.94, 1.96)				
High Cr + Low Cu	108/216	1.45 (1.01, 2.07)*				
High Cr + High Cu	141/221	1.89 (1.33, 2.69)*				
Co-Zn			1.08 (0.66, 1.76)	0.11 (-0.43, 0.64)	0.08 (-0.30, 0.45)	1.36 (0.22, 8.51)
Low Co + Low Zn	96/246	Ref				

Low Co + High Zn	106/238	1.00 (0.70, 1.43)				
High Co + Low Zn	108/236	1.29 (0.90, 1.84)				
High Co + High Zn	123/219	1.40 (0.98, 1.99)				
Co-As			0.98 (0.60, 1.59)	0.06 (-0.56, 0.68)	0.04 (-0.33, 0.40)	1.09 (0.42, 2.84)
Low Co + Low As	102/277	Ref				
Low Co + High As	100/207	1.29 (0.89, 1.86)				
High Co + Low As	95/212	1.36 (0.95, 1.94)				
High Co + High As	136/243	1.71 (1.21, 2.41)*				
Co-Cu			0.82 (0.50, 1.33)	-0.17 (-0.85, 0.51)	-0.10 (-0.48, 0.29)	0.82 (0.40, 1.69)
Low Co + Low Cu	95/265	Ref				
Low Co + High Cu	107/219	1.48 (1.03, 2.12)*				
High Co + Low Cu	103/223	1.49 (1.05, 2.13)*				
High Co + High Cu	128/232	1.80 (1.27, 2.56)*				
Zn-As			1.02 (0.62, 1.66)	0.03 (-0.50, 0.56)	0.02 (-0.38, 0.42)	1.10 (0.19, 6.50)
Low Zn + Low As	90/244	Ref				
Low Zn + High As	114/238	1.26 (0.88, 1.82)				
High Zn + Low As	107/245	1.04 (0.72, 1.48)				
High Zn + High As	122/212	1.33 (0.92, 1.92)				
Zn-Cu			1.03 (0.63, 1.70)	0.05 (-0.48, 0.59)	0.04 (-0.35, 0.43)	1.16 (0.23, 5.84)
Low Zn + Low Cu	109/290	Ref				
Low Zn + High Cu	95/192	1.31 (0.91, 1.87)				
High Zn + Low Cu	89/198	1.03 (0.72, 1.47)				
High Zn + High Cu	140/259	1.39 (1.00, 1.93)				
As-Cu			1.14 (0.70, 1.86)	0.25 (-0.32, 0.82)	0.15 (-0.18, 0.48)	1.58 (0.43, 5.83)
Low As + Low Cu	85/231	Ref				
Low As + High Cu	112/258	1.24 (0.87, 1.78)				

High As + Low Cu	113/257	1.19 (0.82, 1.71)
High As + High Cu	123/193	1.68 (1.15, 2.46)*

Model was adjusted for age, education level, residence area, smoking status, alcohol use, physical activity, sitting time, BMI, diabetes mellitus, hypertension, TC, TG, metals in the multiple-metal model.

[a] OR- int was assessed on the multiplicative scale by including cross-product terms in the model.

[b] RERI, AP and S were assessed on additive scale.

*p <0.05
